# Muscular Power of the Insane

**Published:** 1852-10

**Authors:** 


*Muscular Power of the Insane.* By M. Morel.—A general popular error prevails that the insane are endowed with inordinate muscular power; and this explains why so many persons are brought to the Mareville Asylum fearfully tied and corded. When M. Morel was first appointed to this, he found numerous patients bound up, reputed dangerous, and especially so because of their vociferations. He set them at liberty, without any ill-effect, and attributes much of the violence that had previously occurred to the ill-conduct of the attendants. He agrees with Jacobi, that, as a general rule, the insane exhibit no inordinate muscular power; and some of the patients of almost colossal stature are easily managed by one person. Indeed, the insane, when engaged in manual labor, soon tire, and require frequent repose. If some of them, by exception, work with a feverish activity, and display great strength, the majority are dejected and languid. The persons in whom he has met with the greatest development of muscular power, belong the following ctaegories:—1. Persons of small stature, delicate complexion, and nervous temperament; and especially females who appear exhausted by their cries and agitation. Among such miserable-looking beings, a power of resistance is developed under certain circumstances, which defies the united energies of several attendants; 2. Insane epileptics; 3. Monimaniacs, who are not yet exhausted by the disease or irrational treatment. When their passion is opposed, these persons sometimes manifest a resistance only to be overcome by several attendants.—*Annal. Med. Psych.*

